# Platelet indices and incident hypertension: effect modification by body mass index in a Chinese cohort

**DOI:** 10.7189/jogh.16.04334

**Published:** 2026-09-25

**Authors:** Zekai Chen, Zhen He, Shasha Zhou, Guanlin Chen, Hong Zheng, Yuxian Wang, Weiqiang Wu, Shouling Wu, Youren Chen

**Affiliations:** 1 Department of Cardiology, The Second Affiliated Hospital of Shantou University Medical College, Shantou, China; 2 Research Center of Translational Medicine, The Second Affiliated Hospital of Shantou University Medical College, Shantou, China; 3 Graduate School of the First Clinical Medical School, Hebei North University, Zhangjiakou, China; 4 Department of Cardiology, Kailuan General Hospital, Tangshan, China

**Keywords:** platelet count, mean platelet volume, plateletcrit, platelet distribution width, incident hypertension, body mass index

## Abstract

**Background:**

Routine platelet indices have been linked to blood pressure and vascular risk. However, longitudinal evidence on the association between platelet count (PLT), mean platelet volume (MPV), plateletcrit (PCT), and platelet distribution width (PDW) and incident hypertension remains limited, and it is unclear whether these associations differ according to adiposity. We aimed to examine associations of PLT, MPV, PCT, and PDW with incident hypertension and assess whether these associations vary by body mass index (BMI).

**Methods:**

We analysed 51,191 hypertension-free adults aged ≥18 years from the 2006 baseline examination of the Kailuan Study, with follow-up through 31 December 2023. We measured PLT, MPV, PCT, and PDW from routine full blood counts. We used weighted Cox regression to estimate overall hazard ratios (HRs) and by Chinese BMI categories, adjusting for age, sex, fasting blood glucose, physical activity, and smoking. Restricted cubic splines assessed dose-response relationships.

**Results:**

During a median follow-up of 8.2 years, 29,491 participants developed hypertension (incidence rate = 70.67 per 1000 person-years). Higher PLT (HR = 1.07; 95% confidence interval (CI) = 1.05–1.09) and PDW (HR = 1.03; 95% CI = 1.01–1.04) were associated with greater hypertension risk, whereas MPV showed a modest inverse association (HR = 0.98; 95% CI = 0.96–1.00) and PCT was not significantly associated (HR = 0.99; 95% CI = 0.98–1.01). Absolute incidence rates increased across Chinese BMI categories from 33.59 to 105.13 per 1000 person-years. BMI-stratified analyses suggested heterogeneous patterns, particularly for MPV and PDW. Splines indicated approximately linear positive associations for PLT and PDW, a mild inverse/flat pattern for MPV, and nonlinear associations for PCT.

**Conclusions:**

Routine platelet indices showed modest associations with incident hypertension, with heterogeneous patterns across BMI categories. These findings suggest that platelet-related haematological traits may reflect BMI-dependent differences in hypertension risk, but external validation and formal prediction analyses are needed before clinical use.

Hypertension is a leading cause of cardiovascular morbidity and mortality globally, affecting over one billion individuals and accounting for more than ten million deaths each year [1]. In China, the prevalence of hypertension continues to rise, driven by ageing populations, lifestyle changes, and urbanisation [2]. While traditional risk factors such as age, salt intake, obesity, and genetics have been extensively studied, growing evidence suggests that systemic inflammation and haematological disturbances, including platelet activation, may play critical roles in early vascular changes preceding hypertension onset [3,4].

Beyond their canonical roles in thrombosis and haemostasis, platelets actively participate in vascular inflammation and endothelial dysfunction [5,6] – hallmarks of hypertension pathophysiology. Common platelet indices derived from a full blood count – platelet count (PLT), mean platelet volume (MPV), platelet distribution width (PDW), and plateletcrit (PCT) – reflect aspects of thrombopoiesis, platelet size, and size heterogeneity, and have been linked to higher blood pressure (BP) levels and hypertension [7–11]. Observational studies indicate that higher PLT and MPV are associated with elevated BP and a greater risk of hypertension [7,8,11], whereas Mendelian randomisation (MR) analyses suggest potential causal effects of PLT, PCT, and PDW on BP, with no evidence supporting a causal effect of MPV [9–11]. However, much of the existing literature is limited by cross-sectional designs [11], a focus on continuous BP rather than incident hypertension [7,10,11], or relatively small sample sizes [7–9]. Consequently, rigorous longitudinal studies that evaluate all four indices within a uniform framework in relation to incident hypertension remain scarce.

Adiposity is a plausible effect modifier of platelet-BP relationships. Obesity is accompanied by chronic low-grade inflammation, oxidative stress, and metabolic perturbations [12] that promote platelet priming and activation, and even reprogram the platelet transcriptome [13,14]. Yet few cohort studies have investigated whether adiposity modifies the associations of PLT, MPV, PCT, and PDW with new-onset hypertension. Accordingly, in a large prospective cohort of Chinese adults free of hypertension at baseline, we examined associations between four platelet indices (PLT, MPV, PCT, PDW) and incident hypertension. We tested whether body mass index (BMI) modified these associations. By leveraging readily available haematological biomarkers within an adiposity-aware framework, we aimed to clarify the haematological correlates of hypertension development and to inform practical, scalable risk stratification in the Chinese context.

## METHODS

We followed the STROBE checklist for cohort studies (Checklist S1 in the **Online Supplementary Document**) [15].

### Study design and population

We performed a prospective cohort analysis within the Kailuan study (registration number: ChiCTR-TNC-11001489) [16], an ongoing community-based cohort in Tangshan, China, with biennial follow-ups since 2006 (baseline) (Methods S1 in the **Online Supplementary Document**). We assessed 101,510 participants at baseline. After the exclusion process (Figure S1 in the **Online Supplementary Document**), the final analytic sample comprised 51,191 participants free of hypertension at baseline.

### Exposure assessment

Baseline platelet indices – PLT (×10^9^/L), MPV (fL), PDW (%), and PCT (%) – were obtained as part of the routine full blood count using automated haematology analysers (Sysmex XT-1800i; Sysmex, Kobe, Japan) [17]. To allow comparability across scales, we standardised each index (z-score) and reported regression results per 1-standard deviation (SD) increase. We measured platelet indices at baseline, before hypertension onset, to represent the initial haematological profile. This approach aligns with prospective biomarker analyses that assess whether routinely available baseline markers are associated with subsequent disease risk.

### Outcome ascertainment

We defined incident hypertension during follow-up as any of the following at subsequent examinations or clinical encounters: systolic BP ≥ 140 mm Hg, diastolic BP ≥ 90 mm Hg, physician-diagnosed hypertension, or initiation of antihypertensive therapy. We defined BP as the average of three consecutive seated measurements, obtained at 1–2-minute intervals [18]. Time at risk accrued from the 2006 examination until incident hypertension, loss to follow-up (censored at the date of last contact), or the administrative end of follow-up on 31 December 2023, whichever occurred first.

### Covariates and minimally sufficient adjustment set

We guided covariate selection with a directed acyclic graph constructed before modelling to identify variables needed to block non-causal backdoor paths between baseline platelet indices and incident hypertension (Methods S1 in the **Online Supplementary Document**). The minimally sufficient adjustment set (MSAS) included age, sex, fasting blood glucose (FBG) (measured by automatic analysers: Hitachi 747; Hitachi, Tokyo, Japan) [17], physical activity, and smoking status. We selected these variables *a priori* to minimise confounding [11].

### Statistical analysis

#### Descriptive statistics

For approximately normally distributed variables, we presented the data as mean (SD) and compared them across Chinese BMI categories using analysis of variance. For non-normal variables, we reported the data as median (interquartile range) and compared using the Kruskal–Wallis rank-sum test. We summarised categorical variables as counts (percentages) and compared them using the Pearson χ^2^ test. For ordered categories (*e.g.* physical activity, smoking), we used the Mantel–Haenszel χ^2^ test for linear trend. For visualisation, we plotted Kaplan-Meier curves of hypertension-free survival after dichotomising each platelet index at the overall median; we evaluated differences with log-rank tests.

#### Primary models: weighted Cox regression

Because the proportional hazards assumption for standard Cox models was violated (Table S8 and Methods S1 in the **Online Supplementary Document**), we used weighted Cox regression to obtain average hazard ratios (HRs) that remain interpretable under non-proportional hazards [19]. Models were fit with the ‘coxphw’ package in *R*. Model 1 was the crude model, and model 2 was adjusted for MSAS (*i.e.* age, sex, FBG, physical activity, smoking). We conducted primary analyses in the overall cohort and stratified by Chinese BMI categories [20]: <18.5, 18.5–23.9, 24.0–27.9, and ≥28.0 kg/m^2^.

#### Dose–response and nonlinearity

To assess nonlinearity, we fitted restricted cubic spline (RCS) models using the ‘rms’ package in *R*, with five knots placed at the 5th, 27.5th, 50th, 72.5th, and 95th percentiles of each platelet index. We used the median value of each platelet index as the reference. We assessed nonlinearity by testing the nonlinear spline terms with a Wald χ^2^ test. We adjusted all spline models for age, sex, FBG, physical activity, and smoking status. We presented exposure-response curves with 95% confidence bands, and we repeated RCS analyses within BMI strata.

#### Sensitivity analyses

To assess robustness, we repeated primary models using the World Health Organization (WHO) BMI categories [21] and waist circumference categories based on Chinese criteria [22] (Methods S1 in the **Online Supplementary Document**). We also repeated analyses using original non-standardised platelet indices, excluding participants who developed hypertension before 1 January 2010, and those with baseline high-normal BP, defined as systolic BP 130–139 mm Hg and/or diastolic BP 85–89 mm Hg.

#### Software

We used IBM SPSS Statistics, version 21.0 (IBM Corp., Armonk, New York, USA) and *R*, version 4.3.0 (R Core Team, Vienna, Austria) for all analyses. We fitted weighted Cox models using the ‘coxphw’ package and performed RCS analyses using the ‘rms’ package in *R*. We reported HRs per 1-SD increase, and the statistical significance was set at a two-sided *P*-value <0.05.

## RESULTS

### Baseline characteristics

Among 51,191 adults free of hypertension at baseline, cardiometabolic and behavioural profiles differed across Chinese BMI categories (**Table 1**). The median age was 49.2 years, and 75.2% of the sample were men. With increasing BMI, participants were more often men and had progressively higher systolic/diastolic BP and fasting glucose; the prevalence of diabetes also rose. Smoking became more common with higher BMI, whereas the proportion with high-school education or above declined. Platelet indices showed BMI-related patterns: PLT increased progressively across categories, MPV was modestly lower in overweight, PDW rose slightly, and PCT remained near-constant (Table S9 and Results S1 in the **Online Supplementary Document**). Overall, these distributions indicate less favourable cardiometabolic profiles at higher adiposity alongside a graded elevation in PLT. To assess potential differences related to sample selection, we compared baseline characteristics between the overall baseline cohort and the final analytic sample. Overall, the two populations showed broadly comparable measured baseline characteristics, except for expected differences in baseline BP due to the exclusion criteria.

**Table 1:** Baseline characteristics of participants stratified by Chinese BMI categories*

		BMI (kg/m^2^)				
	**All (n = 51,191)**	**<18.5 (n = 1,323)**	**18.5–23.9 (n = 23,345)**	**24.0–27.9 (n = 19,905)**	**≥28.0 (n = 6,618)**	***P*-value†**
**Age in years**	49.22 (40.77–55.91)	47.72 (31.15–60.29)	48.83 (39.79–55.88)	49.83 (42.13–55.97)	48.53 (39.89–55.43)	<0.001
**Systolic BP, mmHg**	120.00 (110.00–125.00)	110.70 (101.30–120.00)	119.30 (110.00–122.00)	120.00 (110.00–128.70)	120.00 (114.70–130.00)	<0.001
**Diastolic BP, mmHg**	80.00 (70.00–80.00)	71.70 (69.30–80.00)	79.30 (70.00–80.00)	80.00 (72.00–80.00)	80.00 (78.00–80.70)	<0.001
**Height, cm, x̄ (SD)**	167.20 (7.06)	167.65 (7.19)	166.91 (7.02)	167.53 (6.99)	167.16 (7.33)	<0.001
**Weight, kg**	67.00 (60.00–75.00)	50.00 (46.00–53.00)	60.30 (56.00–65.00)	72.50 (68.00–77.00)	84.00 (78.00–89.00)	<0.001
**BMI, kg/m^2^**	24.14 (21.97–26.40)	17.85 (17.60–18.22)	22.03 (20.76–23.03)	25.67 (24.80–26.67)	29.41 (28.62–30.82)	<0.001
**WC, cm**	85.00 (78.30–91.00)	71.00 (66.00–78.00)	80.00 (75.00–85.00)	88.00 (83.00-92.00)	95.00 (90.00-100.00)	<0.001
**PLT, ×10^9^/L**	203.00 (172.00–239.00)	198.00 (166.50–234.50)	202.00 (171.00–239.00)	204.00 (172.00–240.00)	209.00 (175.00–244.00)	<0.001
**MPV, fL**	7.60 (7.00–8.10)	7.70 (7.20–8.20)	7.60 (7.00–8.10)	7.50 (7.00–8.10)	7.60 (7.10–8.10)	<0.001
**PCT, %**	0.16 (0.13–0.20)	0.15 (0.12–0.20)	0.16 (0.12–0.20)	0.16 (0.13–0.20)	0.16 (0.13–0.20)	<0.001
**PDW, %**	13.60 (12.40–15.10)	13.50 (12.30–15.40)	13.50 (12.40–15.10)	13.60 (12.50–15.10)	13.60 (12.50–15.20)	<0.001
**FBG, mmol/L**	5.02 (4.60–5.52)	4.78 (4.40–5.20)	4.96 (4.54–5.40)	5.10 (4.67–5.61)	5.20 (4.72–5.76)	<0.001
**Sex, n (%)**						<0.001
Women	12,718 (24.8)	440 (33.3)	6,699 (28.7)	4,138 (20.8)	1,441 (21.8)	
Men	38,473 (75.2)	883 (66.7)	16,646 (71.3)	15,767 (79.2)	5,177 (78.2)	
**Marital status, n (%)**						<0.001
In a relationship	48,559 (94.9)	1,162 (88.0)	21,998 (94.3)	19,091 (95.9)	6,308 (95.4)	
Not in a relationship	2614 (5.1)	158 (12.0)	1,339 (5.7)	811 (4.1)	306 (4.6)	
**Education level, n (%)**						<0.001
No high school diploma	38,437 (75.1)	883 (67.0)	17,276 (74.1)	15,328 (77.0)	4,950 (74.9)	
High school diploma or higher	12,720 (24.9)	434 (33.0)	6,053 (25.9)	4,570 (23.0)	1,663 (25.1)	
**PA, n (%)**						0.854
None	4,454 (8.7)	97 (7.3)	2,059 (8.8)	1,750 (8.8)	548 (8.3)	
Occasional	39,872 (77.9)	1,063 (80.3)	18,140 (77.7)	15,468 (77.7)	5,201 (78.6)	
Regular	6,865 (13.4)	163 (12.3)	3,146 (13.5)	2,687 (13.5)	869 (13.1)	
**Smoking status, n (%)**						<0.001
Never	30,809 (60.2)	837 (63.3)	14,480 (62.0)	11,613 (58.3)	3,879 (58.6)	
Quit	2,350 (4.6)	45 (3.4)	872 (3.7)	1,084 (5.4)	349 (5.3)	
Occasional	1,963 (3.8)	42 (3.2)	784 (3.4)	838 (4.2)	299 (4.5)	
Daily	16,069 (31.4)	399 (30.2)	7,209 (30.9)	6370 (32.0)	2,091 (31.6)	
**Diabetes, n (%)**						<0.001
No	48,040 (93.8)	1,282 (96.9)	22,305 (95.5)	18,426 (92.6)	6,027 (91.1)	
Yes	3,151 (6.2)	41 (3.1)	1,040 (4.5)	1,479 (7.4)	591 (8.9)	

BMI – body mass index, BP – blood pressure, FBG – fasting blood glucose, MPV – mean platelet volume, SD – standard deviation, PA – physical activity, PCT – plateletcrit, PDW – platelet distribution width, PLT – platelet count, WC – waist circumference, x̄ – mean

*Values are presented as median (interquartile range) unless specified otherwise.

†Categorical variables were compared using the Pearson χ^2^ test, while ordered categorical variables (*e.g.* PA and smoking status) were compared using the Mantel-Haenszel χ^2^ test (linear-by-linear association).

### Time to incident hypertension: Kaplan-Meier estimates

During a median follow-up of 8.2 years, 29,491 participants developed hypertension, corresponding to an overall incidence rate of 70.67 per 1000 person-years. The incidence rate increased progressively across Chinese BMI categories, from 33.59 in underweight participants to 105.13 per 1000 person-years in participants with obesity, indicating a clear absolute-risk gradient by adiposity status.

**Figure 1:**
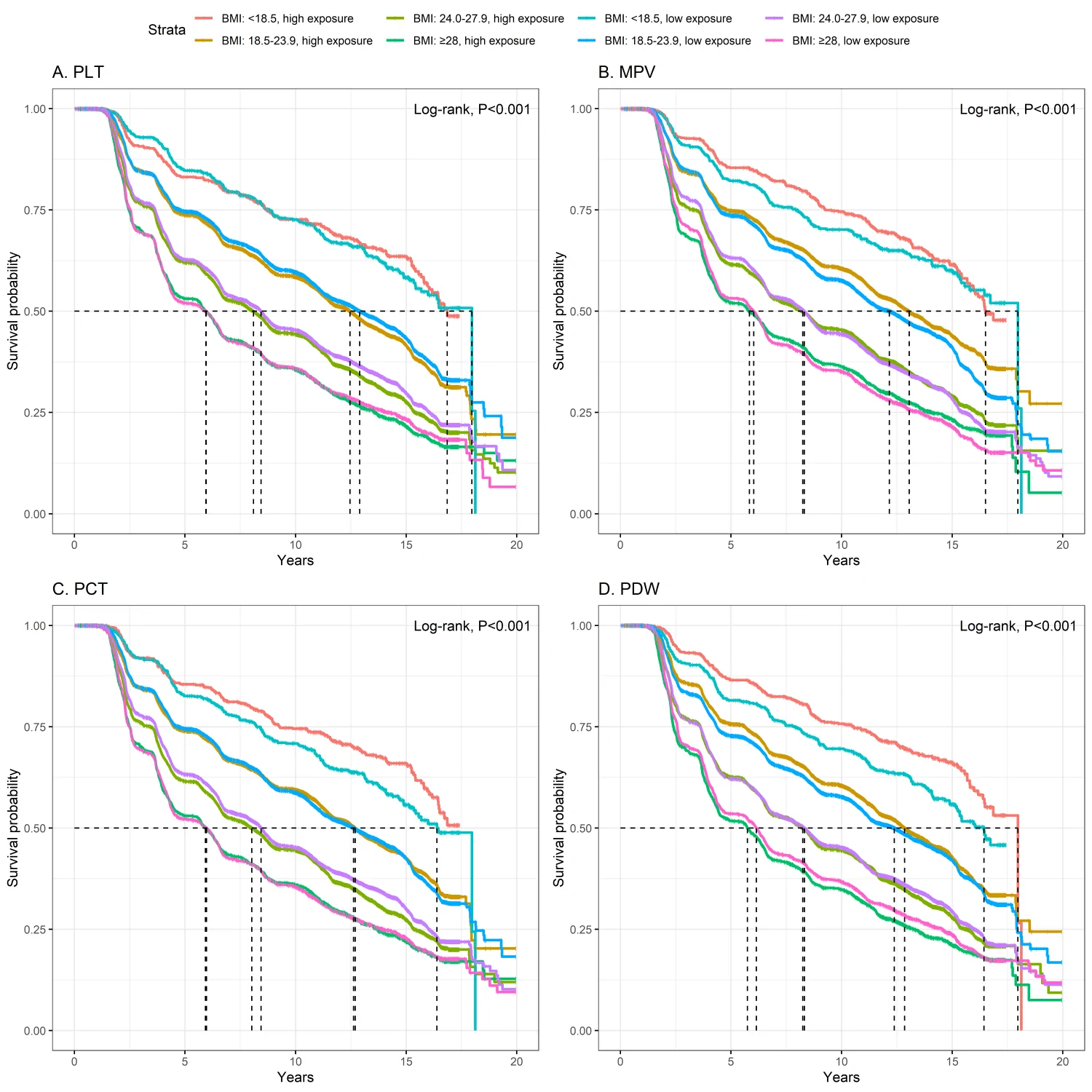
Kaplan-Meier curves for the joint effects of platelet-related traits on incident hypertension. **Panel A.** PLT. **Panel B.** MPV. **Panel C.** PCT. **Panel D.** PDW. High exposure was defined as a value equal to or above the median, and low exposure as a value below the median. BMI – body mass index, MPV – mean platelet volume, PCT – plateletcrit, PDW – platelet distribution width, PLT – platelet count.

Kaplan-Meier curves for time to incident hypertension, with each platelet index dichotomised at the cohort median, showed visually distinct patterns across platelet indices and BMI strata (**Figure 1**). For PLT, higher values were generally associated with earlier hypertension onset, particularly in the normal-weight and overweight groups. For MPV, higher values corresponded to later hypertension onset mainly among normal-weight participants, with little separation in other BMI strata. For PCT, curves overlapped across BMI categories except for a modest separation in the underweight group. For PDW, higher values were associated with earlier hypertension onset in overweight and obese participants, whereas the underweight group showed an opposite pattern (**Figure 1****,** Panels A–D). These visual patterns were broadly consistent with the adjusted model estimates (**Table 2**).

**Table 2:** Weighted Cox regression results for platelet traits and new-onset hypertension

			Exposure*							
			**Total**		**Q2 *vs.* Q1**		**Q3 *vs.* Q1**		**Q4 *vs.* Q1**	
	**Cases, N/n**	**Incidence per 1000 person-years**	**HR (95% CI)**	***P*-value**	**HR (95% CI)**	***P*-value**	**HR (95% CI)**	***P*-value**	**HR (95% CI)**	***P*-value**
**Total**	29,491/51,191	70.67								
PLT			1.07 (1.05–1.09)	<0.001	1.12 (1.06–1.18)	<0.001	1.15 (1.09–1.21)	<0.001	1.22 (1.15–1.28)	<0.001
MPV			0.98 (0.96–1.00)	0.046	0.99 (0.95–1.04)	0.799	0.98 (0.92–1.03)	0.410	0.95 (0.90–1.00)	0.031
PCT			0.99 (0.98–1.01)	0.360	1.13 (1.07–1.19)	<0.001	1.16 (1.10–1.23)	<0.001	1.16 (1.11–1.22)	<0.001
PDW			1.03 (1.01–1.04)	<0.001	1.02 (0.96–1.08)	0.512	1.05 (0.99–1.11)	0.111	1.06 (1.02–1.12)	0.008
**BMI < 18.5**	431/1,323	33.59								
PLT			1.07 (0.96–1.20)	0.200	2.70 (1.22–5.96)	0.014	1.83 (1.00–3.35)	0.049	1.82 (0.99–3.35)	0.054
MPV			0.91 (0.81–1.03)	0.150	0.89 (0.64–1.23)	0.464	1.03 (0.75–1.42)	0.849	0.85 (0.62–1.17)	0.332
PCT			0.90 (0.80–1.01)	0.083	0.97 (0.72–1.32)	0.865	0.88 (0.63–1.22)	0.433	0.94 (0.71–1.25)	0.690
PDW			0.81 (0.70–0.93)	0.003	0.73 (0.55–0.96)	0.027	1.10 (0.74–1.61)	0.645	0.37 (0.19–0.74)	0.005
**BMI 18.5–23.9**	11,592/23,345	56.20								
PLT			1.06 (1.02–1.11)	0.005	1.06 (0.96–1.18)	0.233	1.15 (1.03–1.28)	0.015	1.18 (1.06–1.31)	0.003
MPV			0.95 (0.92–0.98)	0.002	0.91 (0.83–1.01)	0.067	0.89 (0.79–1.00)	0.043	0.84 (0.77–0.92)	<0.001
PCT			1.01 (0.99–1.05)	0.530	1.10 (0.98–1.22)	0.098	1.11 (0.99–1.26)	0.079	1.15 (1.05–1.27)	0.003
PDW			0.997 (0.97–1.03)	0.870	0.99 (0.89–1.11)	0.907	0.98 (0.87–1.08)	0.570	0.98 (0.91–1.06)	0.679
**BMI 24.0–27.9**	12,715/19,905	83.10								
PLT			1.08 (1.05–1.10)	<0.001	1.13 (1.06–1.21)	<0.001	1.12 (1.05–1.20)	<0.001	1.23 (1.15–1.32)	<0.001
MPV			0.999 (0.975–1.02)	0.920	0.99 (0.93–1.06)	0.810	0.99 (0.93–1.06)	0.842	1.01 (0.95–1.07)	0.857
PCT			0.995 (0.977–1.01)	0.600	1.06 (0.99–1.13)	0.098	1.15 (1.07–1.23)	<0.001	1.15 (1.08–1.22)	<0.001
PDW			1.02 (1.00–1.05)	0.024	1.03 (0.96–1.10)	0.396	1.05 (0.98–1.13)	0.146	1.06 (1.00–1.13)	0.058
**BMI ≥ 28**	4,753/6,618	105.13								
PLT			1.03 (0.998–1.06)	0.067	1.07 (0.97–1.17)	0.188	1.06 (0.97–1.16)	0.212	1.07 (0.98–1.18)	0.136
MPV			1.00 (0.97–1.04)	0.900	1.07 (0.98–1.17)	0.114	1.12 (1.01–1.24)	0.024	1.03 (0.93–1.13)	0.602
PCT			1.00 (0.97–1.04)	0.890	1.11 (1.01–1.22)	0.029	1.09 (0.99–1.19)	0.072	1.07 (0.98–1.17)	0.126
PDW			1.06 (1.03–1.10)	<0.001	1.06 (0.97–1.16)	0.172	1.12 (1.02–1.23)	0.019	1.18 (1.08–1.29)	<0.001

BMI – body mass index, CI – confidence interval, HR – hazard ratio, MPV – mean platelet volume, PCT – plateletcrit, PDW – platelet distribution width, PLT – platelet count

*Adjusted for age, sex, fasting glucose, physical activity, and smoking status. The quartiles of PLT indicators are as follows: PLT (172.00, 203.00, 239.00 × 10^9^/L), MPV (7.00, 7.60, 8.10 fL), PCT (0.13, 0.16, 0.20%), and PDW (12.40, 13.60, 15.10%), respectively.

### Primary associations in the overall cohort

**Figure 2:**
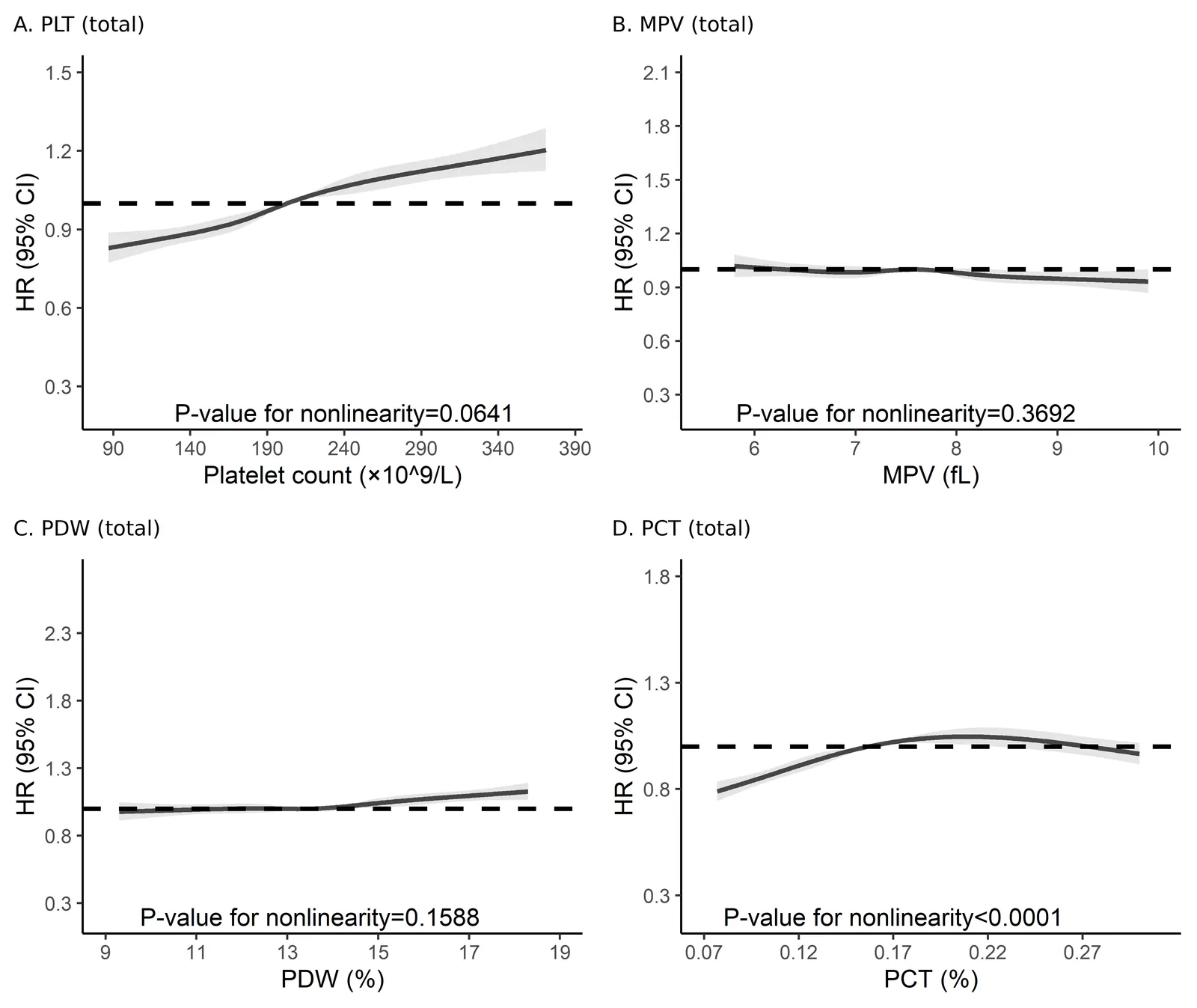
Overall dose-response associations between platelet indices and incident hypertension. **Panel A.** PLT. **Panel B.** MPV. **Panel C.** PDW. **Panel D.** PCT. Models were adjusted for age, sex, physical activity, smoking status, and fasting blood glucose. CI – confidence interval, HR – hazard ratio, MPV – mean platelet volume, PCT – plateletcrit, PDW – platelet distribution width, PLT – platelet count.

Weighted Cox models (model 2) adjusting for the MSAS (*i.e.* age, sex, FBG, physical activity, and smoking) indicated that per 1-SD higher PLT (HR = 1.07; 95% confidence interval (CI) = 1.05–1.09) and PDW (HR = 1.03; 95% CI = 1.01–1.04) were associated with greater risk of incident hypertension, whereas MPV (HR = 0.98; 95% CI = 0.96–1.00) was inversely associated and PCT (HR = 0.99; 95% CI = 0.98–1.01) showed no significant association (**Table 2**). In categorical analyses, all four indices showed significant associations with incident hypertension, each displaying a progressive change in risk across quartiles (increasing for PLT, PDW, and PCT, and decreasing for MPV). The HRs for Q4–Q1 contrasts were 1.22 for PLT, 1.06 for PDW, and 1.16 for PCT, whereas MPV showed an inverse association (HR = 0.95). The crude models (model 1) yielded similar estimates and identical inferences (Table S1 in the **Online Supplementary Document**). The RCS analyses, adjusted for the same MSAS covariates as model 2, further characterised the exposure-response shapes: PLT showed an approximately linear increase in risk (*P* for nonlinearity = 0.06), MPV displayed an essentially flat to mildly inverse profile with no evidence of nonlinearity (*P* = 0.37), PDW exhibited a gentle positive gradient, largely compatible with linearity (*P* = 0.16), and PCT followed a shallow convex (‘hump-shaped’) curve – modest elevation in the mid-range with attenuation at higher values – indicating clear nonlinearity (*P <* 0.001) (**Figure 2****,** Panels A–D).

### Effect modification by BMI

**Figure 3:**
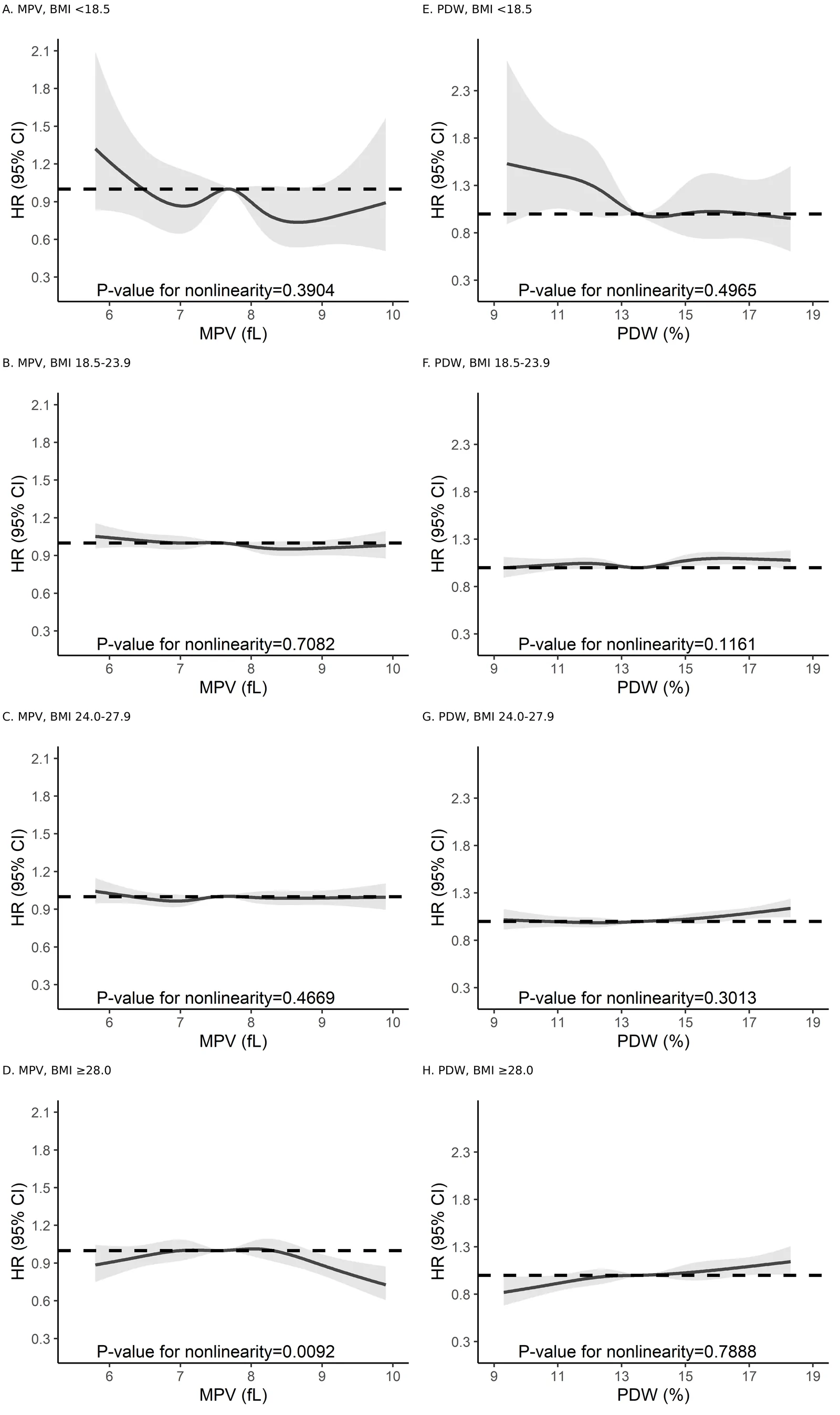
BMI-stratified dose-response associations for MPV and PDW. **Panel A.** MPV, BMI < 18.5. **Panel B.** MPV, BMI = 18.5–23.9. **Panel C.** MPV, BMI = 24.0–27.9. **Panel D.** MPV, BMI ≥ 28.0. **Panel E.** PDW, BMI < 18.5. **Panel F.** PDW, BMI = 18.5–23.9. **Panel G.** PDW, BMI = 24.0–27.9. **Panel H.** PDW, BMI ≥ 28.0. Models were adjusted for age, sex, physical activity, smoking status, and fasting blood glucose. BMI – body mass index, CI – confidence interval, HR – hazard ratio, MPV – mean platelet volume, PDW – platelet distribution width.

Associations differed by adiposity (Chinese BMI categories). There was a positive association between PLT and hypertension across all BMI groups, with attenuation toward borderline in obesity. On the other hand, PDW was positively associated with hypertension risk among overweight and obese participants, whereas the estimate among underweight participants was inverse. An inverse association was found between MPV and hypertension that was confined to normal-weight individuals. We observed positive associations for PCT in normal-weight and overweight groups. The RCS analysis within BMI strata supported these patterns: steeper positive slopes for PLT and PDW in higher BMI strata and a clearer inverse segment for MPV in normal weight (**Figure 3**; Figure S2 in the **Online Supplementary Document**). For PCT, RCS revealed marked nonlinearity, with risk increases confined to the upper range – consistent with null per 1-SD estimates but significant associations in higher quartiles. Interactions between standardised platelet indices and BMI were significant for MPV (*P* = 0.012) and PDW (*P* < 0.001), but not for PLT (*P* = 0.223) and PCT (*P* = 0.989).

### Sensitivity analyses

Sensitivity analyses yielded qualitatively similar inferences when using WHO BMI categories, original non-standardised platelet indices, and early-case exclusion (Tables S2–5 in the **Online Supplementary Document**). After excluding participants with baseline high-normal BP, 39,656 participants remained, and associations were broadly consistent with the primary analysis (Table S6 and Results S1 in the **Online Supplementary Document**). Waist circumference-stratified analyses also supported the main findings: PLT remained positively associated with hypertension, PDW was positive mainly in pre–central and central obesity, MPV was inverse only in normal waist circumference, and PCT remained null on a continuous scale but positive in categorical analyses (Table S7 in the **Online Supplementary Document**).

## DISCUSSION

In this large, community-based Chinese cohort, all four platelet indices were associated with incident hypertension, although with distinct directional patterns. While PLT and PDW showed positive associations with risk, MPV showed an inverse relationship, and PCT showed positive associations in categorical analyses despite null per SD estimates. These relationships varied by adiposity: the PLT-risk association weakened in obesity, the inverse MPV association was largely restricted to normal weight, and the PDW-risk association shifted direction – from inverse in underweight to positive in higher adiposity groups, while PCT showed a pattern of positive associations predominantly in normal-weight and overweight individuals. The RCSs supported these heterogeneous patterns, and sensitivity analyses using alternative adiposity definitions yielded consistent findings.

Our PLT findings align with observational work linking higher PLTs to higher BP and hypertension risk [7,11]. They also align with genetic evidence [9–11]: a multivariable MR study [10] showed that PLT and PDW exert independent effects on BP, whereas MPV and PCT did not after accounting for collinearity across indices. Reverse-MR signals from BP to PLT/PCT were weak [10,11], supporting directionality from platelets to BP. Our PCT findings should be interpreted cautiously because PCT reflects total platelet mass and is closely related to PLT and MPV. Prior MR evidence provides stronger, more consistent support for PLT and PDW than for MPV or PCT, whereas observational estimates may vary by population characteristics, adiposity distributions, BP definitions, and modelling strategies [7–11,23]. Therefore, the positive categorical associations observed for PCT in our study should be viewed as supportive but not definitive evidence, particularly given the null estimates observed when PCT was modelled continuously.

A previous Chinese cohort (n = 9168) with a nine-year follow-up [8] reported a positive MPV-hypertension gradient, whereas our larger cohort showed an overall inverse association limited to normal weight. This discrepancy may reflect differences in model conditioning, particularly adjustment for PLT and baseline systolic BP, exposure coding, and untested adiposity-related effect modification in their study. Because platelet indices are tightly coupled and BMI may mask opposing subgroup effects, our uniform assessment of all four indices and explicit BMI interaction tests provide more comparable evidence. Emerging data also suggest age-dependent heterogeneity for PDW-hypertension associations [23], supporting prespecified context-specific analyses in future studies.

Biologically, platelets actively drive vascular inflammation and endothelial dysfunction, key processes that initiate and maintain hypertension [24]. Contemporary reviews and experimental studies highlight oxidative stress pathways that converge on reduced nitric oxide bioavailability at the onset of hypertension [24,25]. Upon activation, platelets adhere to and signal with leukocytes and the endothelium, form aggregates, release thromboxane A_2_ and serotonin [26], and shed platelet-derived extracellular vesicles [27]. These vesicles carry functional cargo that propagates vascular injury, including NADPH oxidase components such as Nox1 that generate reactive oxygen species [28], adhesion and co-stimulatory proteins such as P-selectin and CD40L [29], procoagulant tissue factor in selected contexts, and regulatory microRNAs (miRs) such as miR-223, miR-21 and miR-34c-5p [30]. Through these mediators, platelets amplify oxidative stress, recruit leukocytes, activate endothelial cells, blunt nitric oxide signalling, and promote small-artery remodelling that sustains elevated BP. These mechanisms provide biological plausibility; however, we did not directly test them in this study. Because we did not measure platelet activation markers, inflammatory cytokines, platelet-leukocyte aggregates, or endothelial function, our results should be interpreted as observational associations rather than evidence for a causal inflammatory or platelet-mediated pathway.

Our BMI-dependent patterns are biologically plausible. Obesity features chronic low-grade inflammation and oxidative stress that prime platelets and heighten endothelial vulnerability [24]. Transcriptional and phosphoproteomic studies show obesity-induced remodelling of platelet gene expression [13] and signalling, with partial normalisation after bariatric surgery [14], supporting a plastic platelet phenotype in obesogenic milieus. Our observation that PDW, a marker of size heterogeneity and indirectly of activation, tracks positively with risk in higher-BMI strata is consistent with obesity-related platelet priming under low-grade inflammation and oxidative stress. In this milieu, higher PDW and PLT plausibly signal greater platelet activation or turnover, accelerating the transition from preclinical vascular dysfunction to overt hypertension through oxidative stress pathways and reduced nitric oxide bioavailability. Higher BMI is also biologically associated with a greater proportion of immature and reactive platelets, as documented in large cohorts [31]. Adipokines may also contribute to these BMI-dependent patterns. Higher BMI is associated with increased leptin and reduced adiponectin, a profile that favours platelet activation and oxidative stress [32]. This adipokine-driven priming may partly explain the stronger associations of PLT and PDW observed in individuals with greater adiposity.

Notably, among underweight participants, the inverse association between PDW and risk may reflect altered thrombopoiesis driven by nutritional deficits or chronic illness rather than lower platelet activation. Nutrient deficiencies such as iron deficiency can shift platelet production and size indices through reactive thrombocytosis [33], while protein-energy malnutrition can alter thrombopoiesis and dampen platelet function [34]. The BMI-stratified findings suggest possible heterogeneity in platelet index-hypertension associations across adiposity groups. Still, they should be interpreted cautiously because multiple platelet indices were examined across several BMI categories. In particular, the inverse association between PDW and incident hypertension among underweight participants should not be overinterpreted, as the underweight stratum was relatively small and the estimate may reflect sparse data, chance variation, or residual confounding. These subgroup findings should therefore be regarded as hypothesis-generating and require confirmation in independent cohorts.

We do not propose platelet indices as stand-alone screening tools or as established additions to hypertension prediction models. The observed associations were modest, and we did not evaluate discrimination, calibration, reclassification, or predictive improvement. Instead, our findings suggest that routinely measured haematological traits may capture BMI-dependent heterogeneity in vascular risk. Formal prediction analyses, external validation, and calibration/discrimination assessments are required before any clinical implementation.

Our study has several strengths. The very large sample and long follow-up, with nearly 30,000 incident hypertension events, enabled precise estimates and stable subgroup analyses. We also used standardised laboratory measurements and prespecified Chinese BMI and waist-circumference strata, supplemented by WHO-based sensitivity checks, thereby improving comparability, relevance to East Asian settings, and analytic robustness. However, several limitations remain. First, despite careful adjustment, we conducted an observational analysis, and residual confounding, such as unmeasured antiplatelet drug exposure, cannot be ruled out. Second, the cohort was predominantly working-age Chinese adults, most of whom were men, which may limit generalisability. Third, the underweight stratum was small, so the inverse PDW association in this group should be cautiously interpreted. Additionally, we modelled platelet indices using measurements from the 2006 baseline examination. Thus, our findings reflect the association of baseline platelet status with subsequent hypertension risk, but not the potential influence of within-person changes in platelet indices over time. Future studies incorporating repeated platelet measurements could clarify whether platelet trajectories provide additional information on hypertension development. Finally, we did not perform phenotype-specific analyses in the present study. Future studies could further explore whether platelet index associations differ across clinically meaningful hypertension phenotypes, such as isolated systolic, diastolic, and systolic-diastolic hypertension.

## CONCLUSIONS

In this large prospective Chinese cohort, routinely measured platelet indices were modestly associated with incident hypertension, with patterns differing across BMI categories. These findings highlight BMI-related heterogeneity in platelet-hypertension associations and support further investigation of platelet-related haematological traits. External validation and formal prediction analyses are needed to determine whether platelet indices add clinically useful information beyond established hypertension risk factors.

## Additional material


Online Supplementary Document

## Data Availability

**Data availability:** The Kailuan cohort data set is not publicly available. Researchers may request access for legitimate scientific purposes by contacting the corresponding authors.
