# Supplementary material for: Platelet indices and incident hypertension: effect modification by body mass index in a Chinese cohort
Source: J Glob Health. 2026 Sep 25;16:04334. doi: 10.7189/jogh.16.04334 (PMC13613294; doi:10.7189/jogh.16.04334)
Supplement: Supplementary file 1 [file jogh-16-04334-s001.zip › jogh-16-04334-s001.pdf]

**Supplement to: Chen Z, He Z, Zhou S, Chen G, Zheng H, Wang Y, Wu W, Wu S, Chen Y. Platelet indices and incident hypertension: effect modification by body mass index in a Chinese cohort. J Glob Health. 2026;16:04334.**


**Supplementary Methods**

**Supplementary Results**

**Figure S1**. Flowchart of participant selection

**Figure S2**. BMI-stratified dose–response associations for PLT and PCT

**Checklist S1 and Tables S1-S9 (.xlsx)**

- **Checklist S1**. STROBE checklist for cohort studies: item-by-item compliance for the present study (.xlsx)
- **Table S1**. Associations of standardized platelet indices with incident hypertension in the overall cohort and by Chinese BMI categories (.xlsx)
- **Table S2**. Associations using original (non-standardized) platelet indices in the overall cohort and by Chinese BMI categories (.xlsx)
- **Table S3**. Associations of standardized platelet indices with incident hypertension in the overall cohort and by WHO BMI categories (.xlsx)
- **Table S4**. Associations using original (non-standardized) platelet indices with incident hypertension in the overall cohort and by WHO BMI categories (.xlsx)
- **Table S5**. Early-case exclusion (removing events before 1 Jan 2010): associations of standardized platelet indices with incident hypertension in the overall cohort and by Chinese BMI categories (.xlsx)
- **Table S6**. Sensitivity analysis excluding participants with baseline high-normal blood pressure (.xlsx)

- **Table S7**. Associations of platelet indices with incident hypertension across waist circumference strata (.xlsx)
- **Table S8**. Schoenfeld residual-based tests of the proportional hazards assumption for standard Cox models (.xlsx)
- **Table S9**. Baseline characteristics of the overall baseline cohort and final analytic sample (.xlsx)

## Supplementary Methods

### Study design and population

The baseline for the present analysis was the 2006 Kailuan examination. All eligible participants entered follow-up at the 2006 examination; later survey waves were used for follow-up and outcome ascertainment but were not used to add new baseline entrants. At each examination, trained staff administered standardised questionnaires (sociodemographic characteristics, lifestyle factors, medical history), conducted physical examinations (including BP and anthropometrics), and collected fasting blood samples for laboratory assays. At baseline, 101,510 participants were assessed. We excluded individuals with missing baseline data on any platelet index (PLT, MPV, PDW, PCT), BP, or BMI; those with prevalent hypertension (self-reported physician diagnosis or antihypertensive treatment); and those with baseline BP ≥140/90 mmHg. We further restricted to adults (≥18 years).

### Covariates and minimally sufficient adjustment set

Age and sex were considered upstream determinants of both haematological traits and hypertension risk. Smoking and physical activity were included because they may influence platelet indices, metabolic status, vascular inflammation, and hypertension development. Fasting blood glucose was included as a marker of glycaemic/metabolic status related to both platelet physiology and hypertension risk. BMI was prespecified as the effect modifier of interest and was examined through BMI-stratified analyses and formal interaction tests. Variables such as lipid profile, alcohol consumption, kidney function, inflammatory conditions, and medication use were considered in relation to the assumed causal structure but were not included in the primary minimally sufficient adjustment set, as they were not required to close confounding paths under the specified DAG. Nevertheless, as in any observational study, residual confounding by incompletely measured cardiometabolic, inflammatory, lifestyle, or medication-related factors remains possible.

Baseline SBP and DBP were not included in the primary DAG-derived adjustment set because baseline BP may represent proximity to the diagnostic threshold or early preclinical hypertension rather than a conventional confounder in this analysis of incident hypertension. PLT, MPV, PCT, and PDW are interrelated measures derived from the same complete blood count, with PCT closely related to PLT and MPV as an indicator of total platelet mass. Therefore, mutually adjusted models were not performed, as they could introduce multicollinearity and change the interpretation of

the estimates. Our findings should be interpreted as index-specific prospective associations rather than independent effects of mutually conditioned platelet traits.

**Operational definitions**

Education was dichotomized as no high school diploma versus high school diploma or higher. Smoking status was categorized as never, former, occasional, or daily smoking according to baseline self-report. Physical activity was categorized as none, occasional, or regular based on self-reported frequency of leisure-time exercise. Marital status was classified as in a relationship versus not in a relationship. Diabetes was defined as fasting blood glucose ≥7.0 mmol/L, self-reported physician diagnosis, or use of glucose-lowering medication.

**Primary models: weighted Cox regression**

Assessment of proportional hazards assumption and weighted Cox regression: We first fitted standard Cox proportional hazards models for each platelet index using the same covariate adjustment set as in the primary analysis. The proportional hazards (PH) assumption was assessed using Schoenfeld residual-based tests. For each model, we examined both variable-specific tests, including the platelet index and covariates, and the global test for the overall model. These tests were used to evaluate whether the association between each covariate and incident hypertension remained constant over follow-up time.

Schoenfeld residual-based tests indicated evidence of non-proportional hazards in all four standard Cox models, with significant global tests for the PLT, MPV, PCT, and PDW models, all $P<2\times10^{-16}$. At the exposure level, the PH assumption was violated for MPV ($P$=$9.1\times10^{-8}$) and PCT ($P$=0.038), but not for PLT ($P$=0.85) or PDW ($P$=0.57).

Several covariates, including fasting blood glucose, age, sex, physical activity, and smoking status, also showed evidence of non-proportional hazards across models. Detailed PH-assumption test results are provided in Table S8 (**Online Supplementary Document**). Therefore, weighted Cox regression was used as the primary modelling approach. This method estimates average hazard ratios, which provide a summary association over follow-up and remain interpretable when the PH assumption is not fully satisfied. The weighted Cox models were fitted using the *coxphw* package in R version 4.3.0, with adjustment for age, sex, fasting blood glucose, physical activity, and smoking status.

**Sensitivity analyses**

To assess robustness, we repeated all primary models using WHO BMI categories (normal weight 18.5–24.9; overweight 25.0–29.9; obesity ≥30.0 kg/m$^2$), applying the same covariate adjustment. We further conducted sensitivity analyses using waist circumference (WC) categories, defined according to Chinese criteria (central obesity: ≥ 90 cm in men and ≥ 85 cm in women; pre–central obesity: 85–89 cm in men and 80–84 cm in women). We additionally conducted an early-case exclusion analysis by removing participants who developed incident hypertension before 1 January 2010 to reduce potential reverse causation and detection bias; effect estimates were then compared with those from the primary analysis to assess consistency in direction and magnitude. To evaluate whether the associations were driven by participants already close to the hypertension diagnostic threshold, we performed a sensitivity analysis excluding participants with baseline high-normal BP, defined as SBP 130–139 mmHg and/or DBP 85–89 mmHg.

## Supplementary Results

### Baseline characteristics

As shown in Table S9 (**Online Supplementary Document**), the distributions of platelet indices, BMI, fasting blood glucose, physical activity, smoking status, and other available baseline characteristics were broadly comparable between the overall baseline cohort and the final analytic sample. The final analytic sample had lower baseline BP, as expected, because participants with baseline hypertension or baseline BP ≥140/90 mmHg were excluded by design. Overall, these comparisons suggest that the final analytic sample was generally similar to the source cohort with respect to measured baseline characteristics.

### Sensitivity analyses

After excluding participants with baseline high-normal BP, 39,656 participants remained. The associations of PLT, MPV, PCT, and PDW with incident hypertension were broadly consistent with those in the primary analysis. In the overall analysis, PLT and PDW remained positively associated with incident hypertension (PLT: HR 1.09, 95% CI 1.07–1.12; PDW: HR 1.04, 95% CI 1.02–1.06), MPV remained modestly inversely associated (HR 0.97, 95% CI 0.95–0.996), and PCT was not materially associated (HR 0.99, 95% CI 0.98–1.00). These findings suggest that the observed associations were unlikely to be driven mainly by participants with high-normal BP at baseline. Detailed estimates are shown in Table S6 (**Online Supplementary Document**).

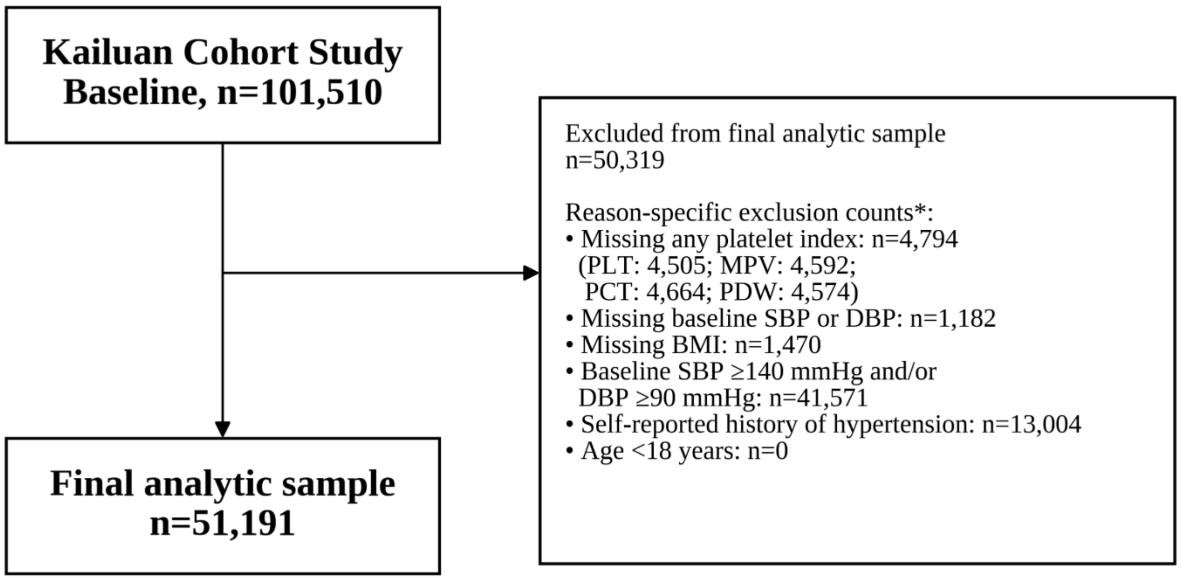


**Figure S1**. Flowchart of participant selection. Because participants could meet more than one exclusion criterion, these reason-specific counts were not mutually exclusive. The final analytic sample comprised 51,191 participants free of hypertension at baseline.

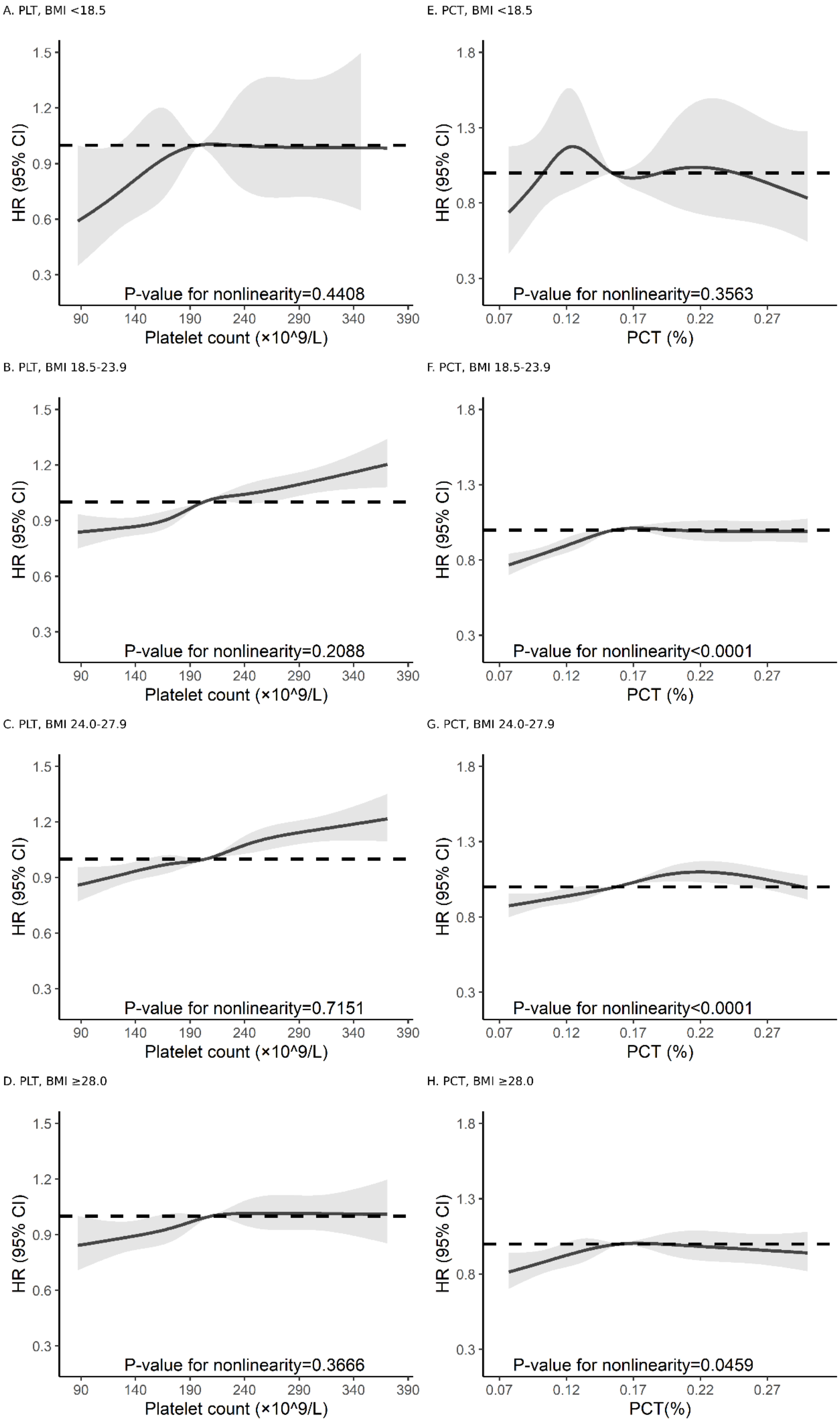

A. PLT, BMI <18.5
HR (95% CI)
P-value for nonlinearity=0.4408
Platelet count (×10^9/L)
E. PCT, BMI <18.5
HR (95% CI)
P-value for nonlinearity=0.3563
PCT (%)
B. PLT, BMI 18.5-23.9
HR (95% CI)
P-value for nonlinearity=0.2088
Platelet count (×10^9/L)
F. PCT, BMI 18.5-23.9
HR (95% CI)
P-value for nonlinearity<0.0001
PCT (%)
C. PLT, BMI 24.0-27.9
HR (95% CI)
P-value for nonlinearity=0.7151
Platelet count (×10^9/L)
G. PCT, BMI 24.0-27.9
HR (95% CI)
P-value for nonlinearity<0.0001
PCT (%)
D. PLT, BMI ≥28.0
HR (95% CI)
P-value for nonlinearity=0.3666
Platelet count (×10^9/L)
H. PCT, BMI ≥28.0
HR (95% CI)
P-value for nonlinearity=0.0459
PCT(%)

**Figure S2**. BMI-stratified dose–response associations for PLT and PCT. **Panels A–D** show PLT across BMI strata: BMI <18.5, 18.5≤BMI<24.0, 24.0≤BMI<28.0, and BMI ≥28.0 $kg/m^2$, respectively. **Panels E–H** show PCT across the same BMI strata. Hazard ratios were estimated using restricted cubic spline models with five knots placed at the 5th, 27.5th, 50th, 72.5th, and 95th percentiles of each platelet index, with the median value as the reference. Models were adjusted for age, sex, physical activity, smoking status, and fasting blood glucose. Shaded areas indicate 95% confidence intervals. BMI – body mass index; CI – confidence interval; HR – hazard ratio; PCT – plateletcrit; PLT – platelet count.
